# Global Establishment Risk of Economically Important Fruit Fly Species (Tephritidae)

**DOI:** 10.1371/journal.pone.0116424

**Published:** 2015-01-14

**Authors:** Yujia Qin, Dean R. Paini, Cong Wang, Yan Fang, Zhihong Li

**Affiliations:** 1 Department of Entomology, College of Agronomy and Biotechnology, China Agricultural University, Beijing, P. R. China; 2 CSIRO Biosecurity Flagship, Canberra, Australia; University of Würzburg, GERMANY

## Abstract

The global invasion of Tephritidae (fruit flies) attracts a great deal of attention in the field of plant quarantine and invasion biology because of their economic importance. Predicting which one in hundreds of potential invasive fruit fly species is most likely to establish in a region presents a significant challenge, but can be facilitated using a self organising map (SOM), which is able to analyse species associations to rank large numbers of species simultaneously with an index of establishment. A global presence/absence dataset including 180 economically significant fruit fly species in 118 countries was analysed using a SOM. We compare and contrast ranked lists from six countries selected from each continent, and also show that those countries geographically close were clustered together by the SOM analysis because they have similar fruit fly assemblages. These closely clustered countries therefore represent greater threats to each other as sources of invasive fruit fly species. Finally, we indicate how this SOM method could be utilized as an initial screen to support prioritizing fruit fly species for further research into their potential to invade a region.

## Introduction

Invasive species can have far reaching ecological and economic impacts worldwide [1–4]. One of the best ways to reduce the likelihood of exotic species invasions is to prevent their establishment, but this relies on being able to initially identify those invasive species with the highest potential to establish [5]. This can be challenging given there are hundreds, or even thousands of species that have the potential to invade and establish in any particular region or country. Many researchers do predict where an invasive species could invade and establish and there are a number of models and approaches used to achieve this. Bioclimatic models in particular, such as CLIMEX, are widely used to predict the potential distribution of an invasive species [6–11]. However, these models require a significant amount of distribution and physiological data, and the model can often take considerable time to develop and validate. As a result, these models usually only assess one or a few species at a time. Subsequently, there is significant need to develop methods which can analyze the hundreds of potentially invasive species simultaneously.

A self-organising map (SOM) [12], which is a type of artificial neural network (ANN), has been used previously to simultaneously rank and prioritize a large number of invasive species by their likelihood to establish in a region. Successful establishment of a species arriving in a new environment depends on both biotic and abiotic factors, which includes climate and, often specific, biotic interactions [13]. The particular combination of species in a region encompasses these complex factors and their interactions, which can be analyzed in a SOM model. In other words, if two regions have a similar assemblages, they are likely to have similar characteristics, and any species present in one of these regions is likely to be able to establish in the other [5, 14]. Thus, a SOM can analyse presence/absence data for hundreds, or even thousands, of species from all regions of the world simultaneously, looking for patterns in species associations, whereby regions with similar suites of invasive species are clustered together and a region-specific likelihood of establishment index (a value between 0 and 1) for each species is generated [14]. In addition, this approach has been shown to be resilient to significant errors in the dataset, which will invariably occur [15], and can accurately rank those species that can establish in a region above those that cannot [14]. Recently, SOM has been applied to a number of different taxa including insect pests [5, 15, 16], fungal pathogens [14], weeds [17], fish [18], and nematodes [19]. A SOM analysis can also identify those regions that have the most similar assemblages and hence present the greatest threat to each other [15, 20].

The family Tephritidae (fruit flies), is among the largest families of Diptera, and includes approximately 4000 species from 500 genera [21]. Currently, it is generally acknowledged that there are probably 1500 fruit fly species relating to fruits; more than 250 species of which are of economic significance [22]. These fruit pest tephritids are found in almost all fruit growing areas of the world [21], where they can cause serious damage to fruit, sometimes resulting in almost total crop failure. *Ceratitis capitata* (medfly) is one species, which is one of the most important worldwide threats to fresh fruits [23], and is capable of infesting from 250 to 400 hosts. For example, this species is estimated to cause US$242 million/year in economic losses in Brazil alone [24]. This example illustrates that some species can become pests in regions far removed from their native range, which is another reason for their economic importance and many fruit fly species have been introduced into new areas by human activities either accidentally or on purpose [25]. In view of the huge damage this group of species can cause, and the fact many have been transported significant distances from their native ranges, many countries focus on them in international trade and enforce quarantine regulations in order to limit their further spread [26–27]. Given this, we generated a dataset of the worldwide distribution of 180 economically significant fruit fly species and analyzed this using a SOM in order to generate ranked lists for each country and identify those species most likely to establish in each region. In addition, we examined how the SOM clustered the countries in order to identify which countries were most similar and hence presented the greatest threat for these fruit fly species.

## Materials and Methods

### Data

We initially extracted the data from the Crop Protection Compendium (CABI 2012), a database associated with most areas in the world encompassing a wide range of different types of information on all aspects of crop protection [28]. The geographical areas represented in the compendium are countries, with many of the large countries further subdivided into states or provinces (e.g. Canada, USA, China, Australia and Brazil). There was distributional data for 55 fruit fly species in this compendium. We combined this data with the distributional data of a further 125 fruit fly species of economic importance extracted from two monographs [21, 25]. Since most of the fruit fly species’ distribution information was not accurate to the states or provinces level we only used country level presence/absence data in this study. Subsequently results of the presence (1) or the absence (0) of each fruit fly species in each geographical area in the database comprised a 118×180 matrix (180 species in 118 countries).

### SOM analysis

A SOM, which is a type of artificial neural network (ANN), is composed of neurons, which compute values from input data, and are arranged in a regular lattice structure. The SOM is used to convert high-dimensional data into a two-dimensional map representing the similarity between data points (in this case, geographic countries). Those data points found close together on the SOM are more similar than those further away [29]. The SOM is therefore a clustering method in which similar data points (in multi-dimensional space) are clustered together in the resultant two-dimensional map [20]. The number of neurons in a SOM is partly determined by the heuristic rule suggested by Vesanto et al. [30], which is 5√n, where n is the number of species. In addition, the two largest eigenvalues are calculated from the data set and the ratio of length and width of the SOM is set to those eigenvalues. Given this ratio, the final number of neurons is set as close to Vesanto’s heuristic rule as possible. The map size used in this analysis was 9×6 (54 neurons) with the standard hexagonal configuration and the recommended number of iterations: 27000 (500 × the number of neurons). Full details describing a SOM analysis can be obtained from [5, 29], but essentially each of the 118 countries occupy a point in multidimensional space, described by the 180-element vector that describes the presence or absence of all 180 fruit fly species in that country. The SOM projects its 56 neurons into this space via neuron weight vectors. As with the region vectors, these neuron weight vectors are composed of 180 elements, which defines the neuron’s location in this multidimensional space. Each SOM neuron therefore occupies a point in the same multidimensional space as the countries, thereby allowing them to ‘interact’ with the country vectors [20].

When the analysis is initiated, each data point is assessed, and the neuron that is closest to this data point in this multidimensional space is identified as the best matching unit (BMU). The neuron weight vector of the BMU is adjusted so that it moves closer to the data point. Because all neurons are connected together similar to a large 9×6 ‘elastic net’, the process of one neuron moving exerts a gravitational force that drags other neurons in the SOM with it. Each country is assessed simultaneously (batch algorithm) to complete one iteration. With the each subsequent iteration, the SOM neurons spread out in the multidimensional space to eventually occupy a similar area to the countries. When the analysis is completed, each country has a BMU, which is its closest neuron. Countries that have similar fruit fly assemblages are located close together in the multidimensional space and will have the same BMU [17].

In this study, the neuron weight vector comprises 180 elements with each element having a value between (0) and (1). Each element corresponds to one of the 180 fruit fly species and indicates how strongly that species is associated with other species in that neuron (BMU) and can be interpreted as a likelihood of establishment [5].

The analysis was performed by using Matlab [31] and the SOM Toolbox (version 2.0) developed by the Laboratory of Information and Computer Science, Helsinki University of Technology (http://www.cis.hut.fi/projects/somtoolbox/). SOM weights were then extracted and used as indices for all fruit fly species for each country in the analysis. Only those species that are known pests of crops grown in a country were included in that country’s list. Crop data was obtained from the FAO [32].

## Results

Establishment likelihood lists of fruit fly species were generated for all 118 countries included in the analysis. Of the 180 fruit fly species, most come from only five genera (Table 1). The top 10 ranked fruit fly species, which are currently absent from each country but have a host present in that country, were extracted (S1) and we present the top ten ranked species for six countries (China, USA, South Africa, Argentina, Italy, and Australia) (Table 2).

**Table 1:** Numbers of fruit fly species in each continent (except for the Antarctic).

	***Anastrepha***	***Bactrocera***	***Ceratitis***	***Dacus***	***Rhagoletis***	**others**	**Total**
**Asia**	0	33	2	8	2	15	60
**Africa**	0	5	12	14	0	13	44
**North America^a^**	26	5	1	0	15	7	55
**South America**	36	0	1	0	6	1	44
**Europe**	0	1	1	0	4	5	11
**Oceania**	0	25	1	3	0	3	32
**world**	44	51	12	19	22	32	180

^a^includes the Central America and Caribbean

**Table 2:** Top ten ranked fruit fly species by establishment index for six countries. Only those species currently absent from a country and a known pest of a host commercially grown in that country were included (for full list see S1 Table).

**China**	**SOM Index**	**South Africa**	**SOM Index**	**The United States**	**SOM Index**
***Bactrocera albistrigata***	0.58	*Bactrocera cucurbitae*	0.55	*Anastrepha obliqua*	0.83
***Bactrocera carambolae***	0.48	*Dacus momordicae*	0.41	*Anastrepha striata*	0.79
***Bactrocera umbrosa***	0.48	*Ceratitis anonae*	0.39	*Anastrepha bezzii*	0.60
***Bactrocera zonata***	0.40	*Acanthiophilus helianthi*	0.21	*Anastrepha pickeli*	0.58
***Bactrocera papayae***	0.37	*Dacus telfaireae*	0.20	*Anastrepha antunesi*	0.52
***Adrama determinata***	0.37	*Dacus humeralis*	0.20	*Anastrepha grandis*	0.51
***Bactrocera arecae***	0.29	*Bactrocera latifrons*	0.11	*Anastrepha leptozona*	0.50
***Monacrostichus citricola***	0.29	*Dacus vansomereni*	0.10	*Anastrepha macrura*	0.45
***Ceratitis capitata***	0.20	*Bactrocera zonata*	0.09	*Anastrepha sororcula*	0.45
***Bactrocera musae***	0.19	*Ceratitis malgassa*	0.08	*Anastrepha rheediae*	0.38
**Argentina**	**SOM Index**	**Italy**	**SOM Index**	**Australia**	**SOM Index**
***Anastrepha obliqua***	0.74	*Rhagoletis cingulata*	0.30	*Bactrocera latifrons*	0.75
***Anastrepha serpentina***	0.69	*Carpomya pardalina*	0.21	*Bactrocera tau*	0.72
***Anastrepha striata***	0.65	*Rhagoletis indifferens*	0.08	*Bactrocera dorsalis*	0.58
***Anastrepha distincta***	0.62	*Dacus ciliatus*	0.06	*Bactrocera caudata*	0.56
***Anastrepha manihoti***	0.47	*Bactrocera invadens*	0.03	*Dacus longicornis*	0.54
***Toxotrypana curvicauda***	0.43	*Dacus frontalis*	0.02	*Bactrocera carambolae*	0.48
***Anastrepha sororcula***	0.41	*Ceratitis cosyra*	0.02	*Bactrocera umbrosa*	0.48
***Anastrepha bezzii***	0.37	*Ceratitis quinaria*	0.02	*Bactrocera zonata*	0.40
***Anastrepha antunesi***	0.33	*Ceratitis rosa*	0.02	*Bactrocera pedestris*	0.39
***Anastrepha leptozona***	0.31	*Bactrocera zonata*	0.01	*Bactrocera papayae*	0.39

China and the US are presented as they have the two largest economies in the world and have been recently identified as central hubs in the agro-food trade network [33]. We also present four countries from each of the four remaining continents (South Africa, Argentina, Italy, and Australia). The top ten fruit fly species currently absent in the US were all *Anastrepha* spp while Argentina had nine species from this genus. The US and Argentina shared the same six *Anastrepha* species in their top ten, and *Anastrepha obliqua* was the top ranked species in both countries. *Bactrocera* dominated both the China and Australia lists, and they shared three same species. It’s noteworthy that the top five species in the Australia list are all present in China. For both South Africa and Italy, the top ten lists were not dominated by any genera.

**Figure 1:**
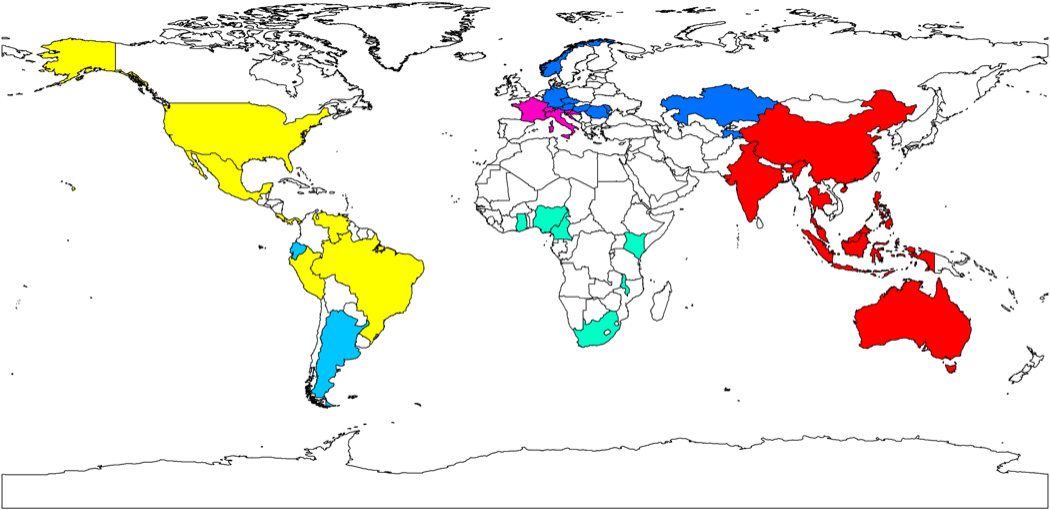
Countries clustering based on fruit fly species assemblages. Map of world showing those countries that were allocated to the same neuron in a SOM analysis (same colour) and hence those countries that have the most similar fruit fly species assemblages.

We also examined how the SOM clustered the countries identifying which countries have the most similar fruit fly assemblages. All 118 countries were clustered into 35 neurons (S2 Table). We noted that many of the countries clustered together by the SOM analysis were also geographically close to each other (Fig. 1). This included North and South American countries clustered together, many Mediterranean countries of Europe clustered together, some sub-Saharan African countries, Australia and Southeast Asian countries clustered with China and India.

## Discussion

While the family Tephritidae’s natural range extends to all world regions, the natural ranges for major pest genera within this group are restricted to particular regions, which explains some of the patterns we found in the SOM analysis. For example, *Anastrepha* spp. are found throughout South and Central America as well as the West Indies, explaining their dominance in the Argentina list as well as their presence in the geographically connected USA. Further, *Bactrocera* spp. are native to tropical Asia, Australia and the South Pacific regions [21], which explains their dominance in the China and Australia lists.

In Africa, most species which attack commercially grown fruit crops belong to *Ceratitis* and *Dacus*, as well as some *Bactrocera* species[34], and as a result, these three genera were found in the South African list (Table 2 and S1 Table). *Rhagoletis* spp have been found throughout the temperate areas of Europe, and the top one in the Italy list belong to this genus. Interestingly, *Anastrepha obliqua*, the species once established in Florida, which triggered a large fruit fly survey and eradication campaign in the 1930s [28, 35], is ranked first in both lists of the US and Argentina. The high ranking of this last species, which previously established in the US, confirms the SOM’s ability to identify those species most likely to establish in a region.

It is interesting to note that those countries geographically close have also been clustered together by the SOM analysis. This is not surprising, as neighbouring countries are more likely to have similar climatic characteristics and are more likely to have shared fruit fly species because of their proximity to each other. However, this also means that a species present in a country will be of greater threat to the neighbouring country if they are found in the same SOM neuron. This is because they not only are likely to have similar environmental characteristics and similar crops, but being geographically close makes it easier for a species to find a pathway. For example, there is a significant amount of trade between India and China, but the highly ranked species for China, *Bactrocera albistrigata, B. carambolae and B. zonata*, are all present in India, where they are recognized as serious pests of tropical and subtropical fruits [28].

There are a number of points that should also be considered with this analysis. Generally, for large countries such as Australia, China and USA it would be preferable to utilise species distributions at their respective states and territories, but this level of data was not available. Secondly, it is likely that a number of errors in distribution are present in our database, given its size, but the SOM has been shown to be resilient to an error rate in species distributions of up to 20% across all countries [15].

The SOM index is a measure of the strength of association of a species with the assemblage of species in a country. Generally, a species that is widespread will have a high SOM index for a particular country. This is unsurprising as this is the quantitative equivalent of asking if a species has invaded another location [17], which is a common question in risk assessments (e.g. [36, 37]) and can indicate the environmental tolerances of an invasive species, and/or its ability to utilise a wide range of hosts/resources. For example, *Ceratitis capitata*, which is currently found in 61 countries, was ranked in the top 5 for 29 countries in which it is not currently found and who grow hosts that could be utilised by this species (S1 Table). Clearly, this extremely polyphagous [38] and highly invasive species represents a significant threat to those countries in which it has not yet invaded.

The SOM is a statistical approach to predicting likelihood of establishment and can be used to identify the most suitable ‘source’ locations for species [15], but it does *not* evaluate the likelihood of a species arriving into a country. Further, the SOM approach does not provide a measure of the impacts from a species. For fruit flies, each major pest genus has a typical pattern of host relationships, most *Rhagoletis*, *Dacus* and *Bactrocera* (*Zeugodacus*) spp. show a strong preference for attacking species of a single plant family, while *Anastrepha*, *Bactrocera. (Bactrocera)* and *Ceratitis* spp. are polyphagous, attacking plants belonging to a wide range of families [21]. This SOM analysis should only be regarded as a preliminary review of biosecurity risk [19], and an assessment of arrival likelihood as well as potential impact would augment the rankings presented here.

A SOM analysis could be used as an initial screening process to reduce the large numbers of potential invasive species to a more manageable number. Moreover, a SOM analysis could be incorporated into an expert elicitation process, by providing the experts with an objective assessment of each species’ establishment likelihood [14]. In this study, we have identified those fruit fly species most likely to establish in six countries and how the world is clustered in terms of fruit fly assemblages. The SOM indices for fruit fly species currently absent from a country could be used to guide debate on which species should be listed for national surveillance needs to achieve early warning. More importantly, the SOM indices could provide a first screen of the fruit fly species prior to a more complete risk assessment [17].

## Supporting Information

S1 Table SOM indices of fruit fly species for each country (species only included if absent from a country and a known pest of a commercially grown host in that country). (XLSX)

S2 Table The countries assigned to the same neuron in the SOM analysis (i.e. those countries with the most similar assemblages). (XLSX)
